# Etude du profil épidémio-clinique du cancer du col utérin au Maroc: cas de la région du Gharb

**DOI:** 10.11604/pamj.2023.45.114.38344

**Published:** 2023-07-04

**Authors:** Mohamed Zraidi, Mohammed Ibriz

**Affiliations:** 1 Plant, Animal and Agro-Industrial Production Laboratory, Department of Biology, Faculty of Sciences of Kenitra, Ibn Tofail University, Kenitra, Morocco University Campus, Kenitra, BP 133, Morocco,; 2 Reference Centre for Screening and Early Diagnosis of Breast and Cervical Cancer, Kénitra Provincial Hospital, Kenitra, Morocco

**Keywords:** HPV, vaccination, dépistage, lésions précancéreuses, cancer du col utérin, prise en charge

## Aux éditeurs de Pan African Medical Journal

Le cancer du col utérin (CCU) est considéré comme un problème de santé publique. Il est placé au quatrième rang des cancers qui causent le plus de décès dans le monde. Selon les statistiques globales du cancer, on a noté 604 000 nouveaux cas et 342 000 décès en 2020 [1,2]. La survenue de cette tumeur est étroitement liée à une infection persistante virale à Papillomavirus (HPV). Au Maroc, Il se classe au deuxième rang, après le cancer du sein chez les femmes, en termes d'incidence et de mortalité.

D'un autre côté, le nombre de nouveaux cas est passé de 2258 en 2012 à 3388 nouveaux cas par an en 2018 et 2500 décès ont été déclarés en 2020 [3]. Le CCU se classe parmi les trois principaux cancers affectant les femmes de moins de 45 ans dans 146 des 185 pays [4]. L'Organisation mondiale de la Santé (OMS) a lancé en 2020, une stratégie d'élimination du CCU d'ici 2030. Il a donc incité chaque Etat membre de s'inscrire dans cette initiative pour éliminer progressivement le CCU et exécuté à la lettre les stratégies suivantes: vaccination contre le HPV, le dépistage des lésions précancéreuses intra-épithéliales et leur traitement, le diagnostic précoce et le traitement approprié du CCU [5,6]. Selon Ferlay J *et al*.[7], le taux de survie à 5 ans du cancer du col utérin métastatique est de 16,5% contre 91,5% pour le cancer du col utérin localisé. Les lésions précancéreuses peuvent être traitées par cryothérapie, thermo-coagulation, résection à l'anse diathermique, chirurgie au LLETZ ou par laser [8]. L'objectif de cette étude consiste en l'évaluation des examens et prise en charge des lésions précancéreuses ou CCU chez des femmes dans la région du Kenitra (Gharb du Maroc).

L'étude a porté sur 297 femmes âgées de 22 à 90 ans. L'âge moyen était de 51,98 ±11,68 ans. La distribution des âges répond aux conditions de la loi de gaussienne (coefficient d'asymétrie= 0,32: coefficient d'aplatissement=0,18), 74% de ces malades sont mariées et 9% sont divorcées, 16% sont veuves et 1% sont célibataires. A peu près 53,88% sont d'origine rurale et 46,12% sont de provenance urbaine. La parité moyenne est de 4,22±0,14, avec un minimum de 0 et un maximum de 15. Toutefois, 39,4% souffrent d'un arrêt de cycle menstruel, 31,9% présentent un cycle irrégulier et 28,6% présentent un cycle régulier. En effet, 76,3% bénéficient d'une couverture dont 56,4% (n=145) des malades disposent d'un RAMED (Régime d'Assistance Médicale aux Economiquement Démunis), 9,7% (n=25) se sont servis d'un CNSS (Caisse Nationale de Sécurité sociale), 5,4% (n=14) bénéficient d'un CNOPS (Caisse Nationale des Organismes de Prévoyance Sociale) et 1,9% (n=5) bénéficient d'un AMO (Assurance Maladie Obligatoire). Cependant 23,7% de ces femmes malades ne bénéficient d'aucune couverture sociale. En tout 54,3% des femmes malades ont répondu qu'elles n'avaient pas fait le FCV (Frottis cervico vaginal).

**Figure 1:**
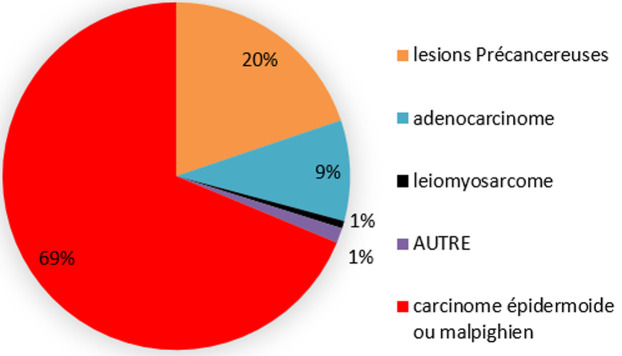
répartition des malades en fonction du résultat de l'examen anatomopathologique

La répartition des femmes malades en fonction de l'examen anatomopathologique montre que (Figure 1) 69% (n=198) ont un carcinome épidermoïdes; 20% (n=57) des patientes ont présenté des lésions précancéreuses (CIN); 9% (n=27) des malades ont présenté un adénocarcinome; 1% (n=2) de patientes ont eu un leiomyosarcome et 1% autres types histologiques. Dans notre échantillon, 87,1% (n=242) des femmes atteintes d'un cancer du col de l'utérus sont adressées à l'institut national d'oncologie (INO), ce dernier les prend en charge. Cinq pour cent (5%) de ces malades sont adressées pour une conisation. Toutefois, 2,9% (n=8) sont adressées à l'INO pour une hystérectomie, une radiothérapie et/ou chimiothérapie et/ou chirurgie.

Le Tableau 1 présente les résultats de la régression linéaire (variable dépendante le grade du cancer) en fonction des variables ci-dessous. Seules les variables mode de couverture sociale et l'âge de la première grossesse ayant montré une association significative avec le grade de la dysplasie avec respectivement une p-value de 0,027 et 0,049.

**Tableau 1:** analyse de la régression simple (variable dépendante)

Modèle		Coefficients non standardisés		Coefficients standardisés	t	Sig.
		B	Erreur standard	Bêta		
1	(Constante)	9,141	1,834		4,983	,000***
	Âge - catégorie	-,038	,035	-,130	-1,084	,280
	Parité	,240	,147	,162	1,632	,105
	Mode de couverture sociale	-,430	,193	-,186	-2,231	,027*
	Âge 1^ere^ grossesse	,541	,150	,101	1,265	,049*
	Cycle menstruel	-,350	,699	-,057	-,500	,618

Au Maroc, en 2018 le nombre de nouveaux cas/an est de 3388 et 2500 décès ont été déclarés en 2020. En France, il occupe le 7^e^ rang, avec 3500 nouveaux cas par an avec une incidence de 9,9/100000 femmes; aux Etats-Unis, le cancer invasif du col de l'utérus est 3 fois moins fréquent que le cancer in situ (CIS) avec une fréquence de 5% des cancers chez la femme [4]. Au Maroc, l'incidence brute est de 11,6 pour 100000 femmes avec un taux de mortalité qui dépasse 50% alors qu’en Tunisie l'indice brut est de 5,7 avec presque le même taux de mortalité alors que cet indice est très faible aux pays du Golf (KSA = 2,4 et UAE =4). Pour ce qui des lésions précancéreuses nous avons trouvé 41% des cas de CINI, 34% de CIN III et 25% de CIN II, selon Schiffman [9].

On observe un pic de survenue de CIN 3 entre 25 et 30 ans. Les études épidémiologiques montrent que l'âge des patientes croît régulièrement avec le degré de la dysplasie, ce qui indique une évolution dans le temps [10]. D'après le même rapport de l'OMS, en 2020, 75 % des femmes atteintes étaient décédées à cause de ce cancer dans les pays à PIB mondial très faible. L'Afrique à elle seule a totalisé, en 2002, 582 000 personnes atteintes de cancer et 412 300 décès durant la même période [2].

**Conclusion:** le Maroc tant que pays en voie de développement, connait un progrès considérable dans le dépistage et le traitement du cancer du col utérin. Bien que le Maroc se soit intégré dans la lutte contre cette maladie, en répondant au programme élaboré par l'OMS, il reste à instaurer le programme de la prévention primaire basée sur le vaccin anti-HPV et l'introduction de test génomique HPV dans le dépistage qui permettrait de réduire les faux positifs et les traitements inutiles par rapport à la stratégie actuelle basée sur l'IVA (inspection visuelle à l'acide acétique). La question de la faisabilité de mise en œuvre et du coût-bénéfice de cette option reste à déterminer.

## References

[1] Bray F, Ferlay J, Soerjomataram I, Siegel RL, Torre LA, Jemal A (2018). Global cancer statistics 2018: GLOBOCAN estimates of incidence and mortality worldwide for 36 cancers in 185 countries. CA Cancer J Clin.

[2] UICC Global Cancer Control GLOBOCAN 2020: New Global Cancer Data.

[3] Gätje R, Eberle C, Scholz C, Lübke M (2011). Kurzlehrbuch Gynäkologie und Geburtshilfe.

[4] Belglaiaa E, Mougin C (2019). Le cancer du col de l'utérus: état des lieux et prévention au Maroc. Bull Cancer.

[5] Naucler P, Ryd W, Törnberg S, Strand A, Wadell G, Elfgren K (2007). Human papillomavirus and Papanicolaou tests to screen for cervical cancer. N Engl J Med.

[6] World Health Organization (WHO) Cervical cancer.

[7] Ferlay J, Steliarova-Foucher E, Lortet-Tieulent J, Rosso S, Coebergh JW, Comber H (2013). Cancer incidence and mortality patterns in Europe: estimates for 40 countries in 2012. Eur J Cancer.

[8] CNLC (2007). Comité National de Lutte contre le cancer. Guide d'information.

[9] Schiffman MH (1992). Recent progress in defining the epidemiology of human papillomavirus infection and cervical neoplasia. J Natl Cancer Inst.

[10] Bouhadef A, Asselah F, Boudriche N, Chaoui F/Z, Benceraie A, Kaddori-Slimani L (2016). Cytopathologie de dépistage des precurceurs et du cancer du col de l'utérus, édition Maquette infographie et publication ADNS.

